# Contribution of the Argonaute-1 Isoforms to Invertebrate Antiviral Defense

**DOI:** 10.1371/journal.pone.0050581

**Published:** 2012-11-29

**Authors:** Tianzhi Huang, Xiaobo Zhang

**Affiliations:** Key Laboratory of Conservation Biology for Endangered Wildlife of Ministry of Education, Key Laboratory of Animal Virology of Ministry of Agriculture and College of Life Sciences, Zhejiang University, Hangzhou, China; University of British Columbia, Canada

## Abstract

Argonaute (Ago) protein, the central component of the RNA interference (RNAi) pathway, plays important roles in host innate antiviral immunity. Most organisms harbor a large number of different Ago proteins and isoforms; however, the roles of Ago isoforms in immune defense against pathogens remain unclear. In the present study, three Argonaute-1 (Ago1) isoforms, termed Ago1A, Ago1B, and Ago1C, were found in *Marsupenaeus japonicus* shrimp. Quantitative real-time PCR (polymerase chain reaction) revealed that isoforms Ago1A and Ago1B containing an insertion sequence in the PIWI domain, were significantly up-regulated in lymphoid organ and hemolymph, and also upon white spot syndrome virus (WSSV) challenge, indicating the involvement of Ago1A and Ago1B in antiviral immunity. The results showed that silencing of Ago1A with a sequence-specific siRNA led to a significant increase of WSSV loads. It was revealed that knockdown of Ago1B mRNA by 37–70% resulted in higher virus loads in shrimp. However, upon silencing Ago1B by more than 85%, a two-fold increase in Ago1A mRNA was observed but viral load was the same as untreated controls challenged with WSSV, suggesting that the simultaneous up-regulation of Ago1A might compensate for the loss of Ago1B. These data indicated that Ago1A played more important roles in the antiviral immune response than Ago1B. The simultaneous inhibition of Ago1A and Ago1B resulted in a greater increase in viral loads than Ago1A or Ago1B alone, indicating that Ago1A and Ago1B isoforms were involved in shrimp antiviral immunity. It was revealed that Ago1C had no effect on virus infection. Therefore, the current study presented the first report on the contribution of Ago isoforms in the invertebrate defense against virus infection.

## Introduction

RNA interference (RNAi) has emerged as an important regulatory mechanism whereby small noncoding RNAs post-transcriptionally control the expression of target genes through mRNA destabilization and translation repression or control gene transcription via chromatin modification [1]. Small noncoding RNAs mainly contain small interfering RNAs (siRNAs) and microRNAs (miRNA), which are generated from double stranded RNA (dsRNA) precursors by an RNaseIII enzyme called Dicer [2], [3]. Processed small RNAs are incorporated into the RNA-induced silencing complex (RISC) where one strand of the duplex is preferentially retained and the other (passenger strand) is discarded [4], [5]. The RISC is guided by the retained RNA strand to a cognate target mRNA, where an Argonaute protein (Ago) mediates translational inhibition/mRNA destabilization by binding to the 3′ untranslated region (UTR), sequence-specific cleavage of the corresponding mRNA or transcriptional silencing of the target DNA [6], [7], [8]. An important role for RNAi has been demonstrated in the innate immune response against viruses in eukaryotes, especially in invertebrates and plants that lack adaptive immunity and therefore rely solely on innate mechanisms to combat viral infections [1], [8].

To execute their biological functions, small noncoding RNAs require a unique class of proteins from the Argonaute family. Ago protein is the central component of RISC, which provides the platform for target-mRNA binding and the catalytic activity for mRNA cleavage in the RNAi pathway [9], [10]. Ago proteins are typically characterized by piwi-argonaute-zwille (PAZ) and PIWI domains [11]. The PAZ domain forms a nucleic acid–binding pocket for binding small RNAs with characteristic two nucleotide (nt) 3′ overhangs trimmed by RNase III-type enzymes such as Dicer [9], [10], [11]. The PIWI domain has an activity that degrades corresponding RNAs [9], [10], [11]. In plants and invertebrates, Ago-mediated silencing activity is required for small RNA-based antiviral immunity. It has been shown that small RNA-based antiviral immunity is abolished in many species by knockdown of a single Ago protein, including Ago2 of *Drosophila melanogaster* and *Anopheles gambiae*, RDe-1 and C04F12.1 of *Caenorhabditis elegans*, and Ago1 and Ago7 of *Arabidopsis thaliana* [12], [13], [14], [15].

Ago proteins can be divided into the Ago subfamily and Piwi subfamily. Except for the fungus *Schizosaccharomyces pombe* that harbors only one Ago protein, most organisms encode a large number of Ago genes. *D. melanogaster* possesses five Ago genes, humans possess eight, *A. thaliana* possesses 10, and *C. elegans* possesses up to 27 [9], [10], [11]. Recently, it was revealed that multiple isoforms from a single Ago2 gene locus were present in some insect species [16]. It was found that the *D. melanogaster* Ago2 gene locus produced a large number of different transcripts that encoded multiple isoforms with variant glutamine-rich repeats (GRRs) copy numbers [16], [17]. However, the functional significance of Ago isoforms remains unknown. The presence of many members within the Ago family and multiple transcript variants from a single gene locus may indicate diverse biological functions of Ago proteins in many biological processes, including cell proliferation and differentiation, apoptosis, cancer, and immune defense.

**Figure 1:**
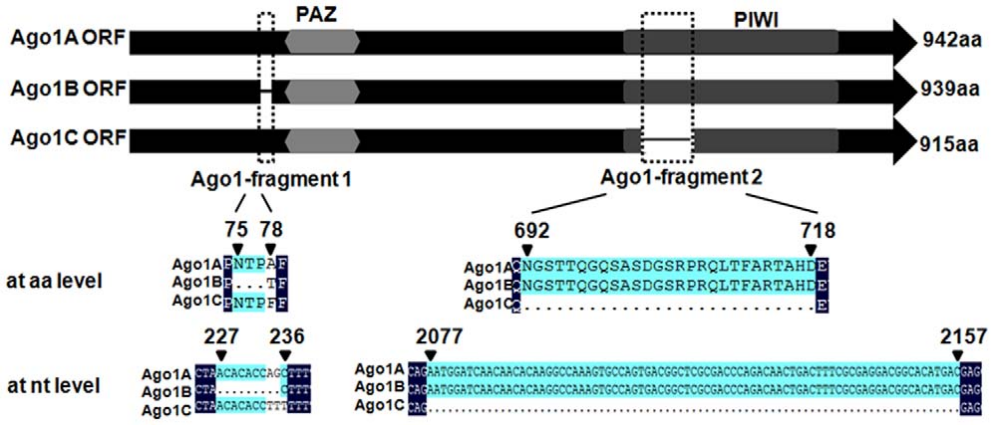
Identification of shrimp Ago1 isoforms. Schematic diagram of three isoforms (Ago1A, Ago1B, and Ago1C) of shrimp Ago1 gene. The numbers show the sites of Ago1-fragment 1 and Ago1-fragment 2 in Ago1.

The present study shows that the shrimp *Marsupenaeus japonicus* possesses three Ago isoforms. This species is an economically important marine invertebrate that, in recent years, has attracted increasing attention as a model for invertebrate-virus interaction. Among three Ago1 isoforms identified, our study revealed that Ago1A and Ago1B that contained an insertion sequence in the PIWI domain were responsible for the host immune response against white spot syndrome virus (WSSV) infection. Therefore, our investigation presented a novel role for Ago isoforms in innate immunity.

**Figure 2:**
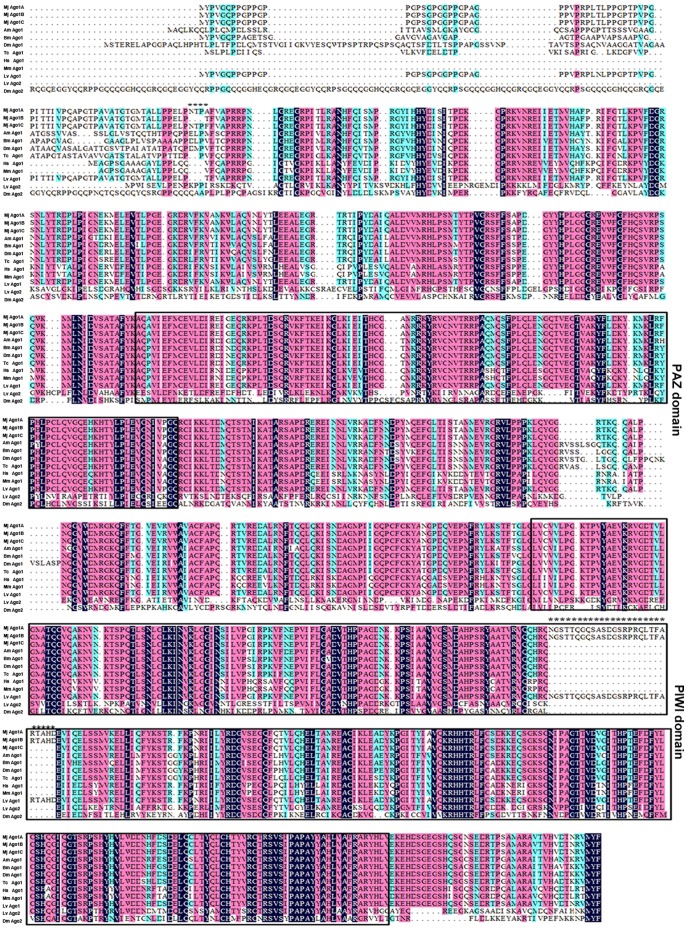
Amino acid alignments of shrimp Ago1 isoforms and Ago homologs from other species. The conserved PAZ and PIWI domains were boxed. Amino acid differences between shrimp Ago1 isoforms were highlighted with asterisks. *Homo sapiens*, Hs Ago1 (GenBank accession no. NP_036331.1); *Mus musculus*, Mm Ago1 (AAI29916.1); *Tribolium castaneum*, Tc Ago1 (XP_971295.2); *Bombyx mori*, Bm Ago1 (NP_001095931.1); *Drosophila melanogaster*, Dm Ago1 (NP_725341.1); Dm Ago2 (NP_Q9VUQ5); *Apis mellifera* Am Ago1 (XP_624444.3); *Litopenaeus vannamei*, Lv Ago1 (NP_ADK25180.1); Lv Ago2 (NP_ADK25181.1); *Marsupenaeus japonicus*, Mj Ago1.

## Materials and Methods

### Shrimp Culture and WSSV Challenge

*M. japonicus* shrimp of approximately 15 g each were raised in groups of 20 individuals in 80 L aquaria filled with air-pumped circulating sea water at 25°C. Three shrimp from each group were randomly selected for WSSV PCR (polymerase chain reaction) detection by WSSV-specific primers (Table S1) to ensure that the shrimp were virus-free before experiments. WSSV-specific primers were used to amplify a region from 226101 to 226401 of the WSSV genome (GenBank accession no. AF332093.1) [18]. Virus-free shrimp were injected with 100 µL WSSV inoculum (10^5^ virus copies/mL) by intramuscular injection using a syringe with a 29-gauge needle [18]. The virus titer was determined by quantitative real-time PCR as described below. At various times post inoculation, shrimp organs or tissues (heart, hemolymph, lymphoid organ, gill, muscle, and hepatopancreas) were collected from three randomly selected specimens and immediately stored in liquid nitrogen. Shrimp assays were carried out in strict accordance with the recommendations in the Guide for the Care and Use of Laboratory Animals of the Zhejiang Province. The protocol was approved by the Committee on the Ethics of Animal Experiments of the University of Zhejiang Univesity, China.

**Figure 3:**
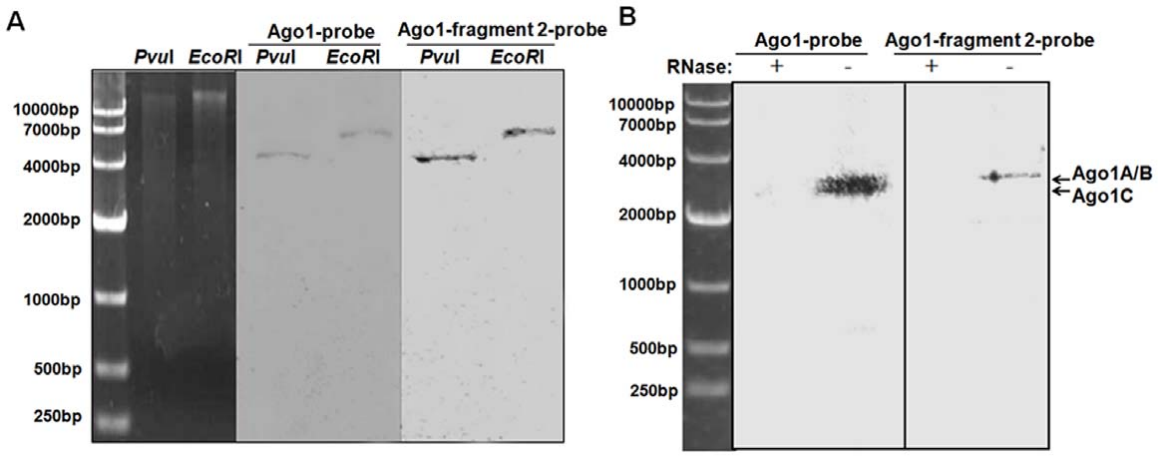
Southern blot and northern blot analysis of shrimp Ago1 isoforms. (**A**) Southern blot of shrimp genomic DNA with DIG-labeled Ago1-probe that could detect three Ago1 isoforms or Ago1-fragment 2-probe that was unique to Ago1A and Ago1B. (**B**) Northern blot of total RNAs extracted from shrimp gills. The probes used were shown on the top. The upper band likely consisted of co-migrated Ago1A and Ago1B transcripts, while the lower band potentially represented the Ago1C transcript.

### RNA Extraction and Complementary DNA (cDNA) Synthesis

Total RNAs were extracted from different tissues or organs of shrimp using the mirVanaP^TMP^ RNA isolation kit according to the manufacturer’s instructions (Ambion, Foster City, USA). To remove any genomic DNA contamination, total RNA extracts were treated with RNase-free DNase I (Takara, Shiga, Japan) at 37°C for 30 min. First-strand cDNA synthesis was performed using 1 µg of total RNA according to the manufacturer’s guidelines for the PrimeScript 1st strand cDNA Synthesis Kit (Takara).

**Figure 4:**
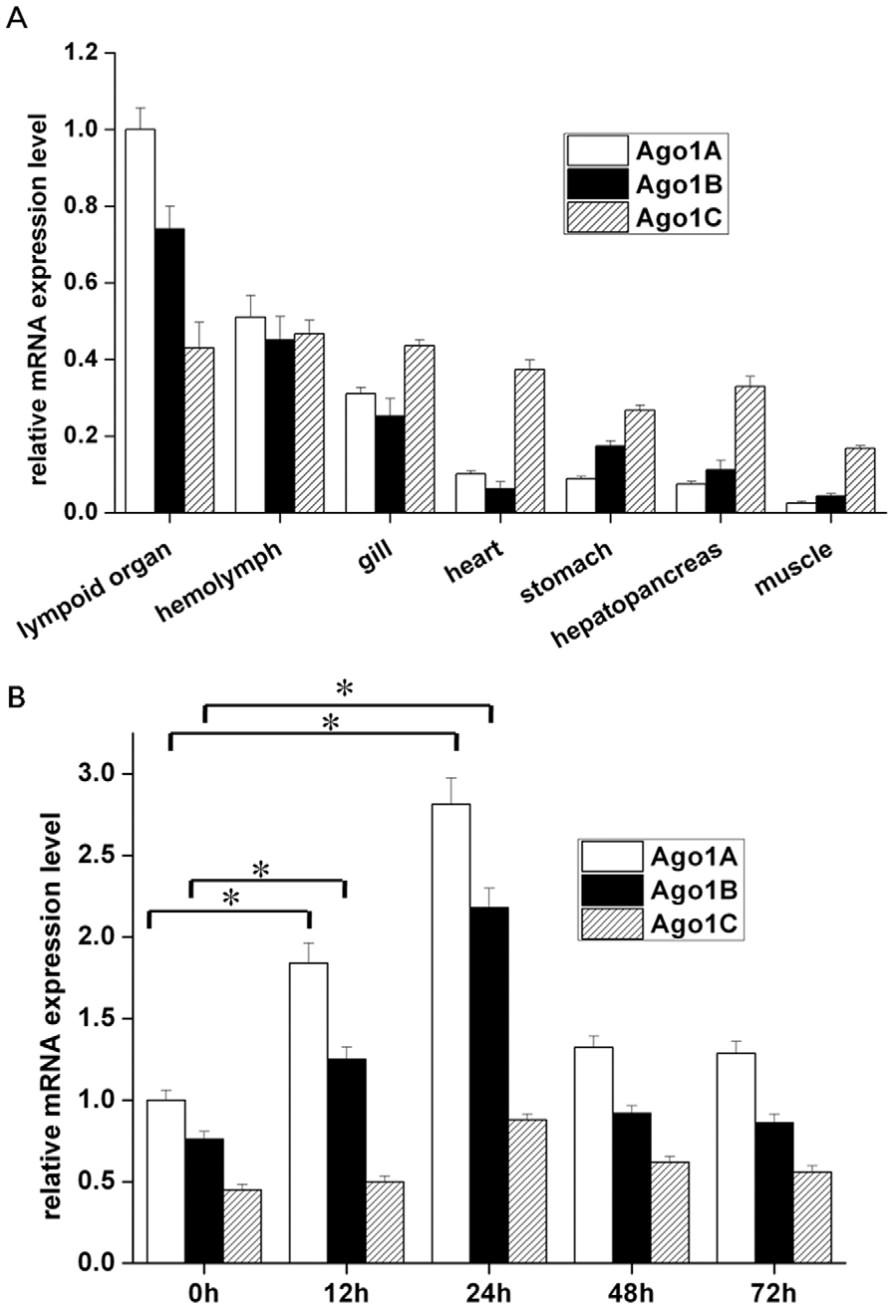
Expression profiles of Ago1 isoforms in shrimp. (**A**) Expression patterns of Ago1 isoforms in different tissues or organs of shrimp as revealed by quantitative real-time PCR. The shrimp β-actin was used as an internal standard. The relative expression levels of Ago1A, Ago1B, and Ago1C mRNAs were compared with that of Ago1A in lymphoid organ. Each column represented the mean of triplicate assays within 1% standard deviation. (**B**) The time-course of expression profiles of Ago1 isoforms in lymphoid organ of shrimp challenged with WSSV by quantitative real-time PCR. The relative expression levels of Ago1A, Ago1B, and Ago1C mRNAs at various times post-inoculation (0, 12, 24, 48, and 72 h) were compared with that of Ago1A at 0 h post-inoculation. The numbers indicated the time points post-inoculation with WSSV. Each column represented the mean of triplicate assays within 1% standard deviation. The statistically significant differences between treatments were represented with an asterisk (*P<0.05).

### Cloning the Full-length cDNA of Shrimp Ago1 Gene

Based on multiple sequence alignments of Ago1 homologs from *D. melanogaster* (GenBank accession no.: NP_725341.1), *Tribolium castaneum* (GenBank accession no.: XP_971295.2) and *Bombyx mori* (GenBank accession no.: NP_001095931.1), degenerate primers (Table S1) matching conserved domains of PAZ and PIWI were used for the partial PCR amplification of the shrimp Ago1 gene. PCR was conducted with an initial denaturation step of 5 min at 94°C, followed by 35 cycles of 94°C for 30 s, 54°C for 40 s and 72°C for 1 min, with a final elongation at 72°C for 10 min. To obtain the full-length sequence of Ago1 cDNA, rapid amplification of cDNA ends (RACE) was performed using a 5′/3′ RACE kit (Roche, Indianapolis, IN, USA). Based on the partial sequence of the Ago1 gene, specific primers (Table S1) for 5′ RACE and 3′ RACE were used for respective RACE PCR and performed according to the manufacturer’s instructions. PCR products were cloned into pMD-18 vector (Takara) and sequenced. After assembling the overlapping fragments, the full-length cDNA of Ago1 was obtained. To confirm the assembled sequence of Ago1 gene, Ago1 cDNA was amplified using Ago1 full-length primers (Table S1) from shrimp lymphoid organs. The PCR protocol used was 94°C for 5 min, followed by 35 cycles of 94°C for 40 s, 54°C for 45 s and 72°C for 3.5 min, with a final elongation at 72°C for 10 min. The resulting PCR products were cloned into pMD-18 vector (Takara) and sequenced.

**Figure 5:**
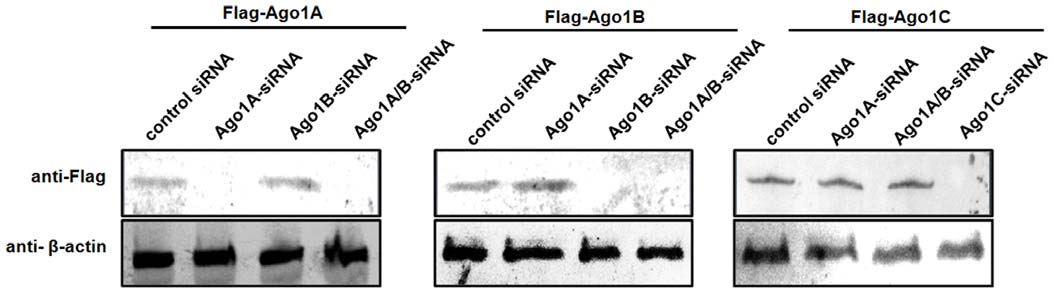
Specificities of siRNAs targeting Ago1 isoforms. S2 cells were transiently co-transfected with the Flag-tagged Ago1 isoform constructs and the isoform-specific siRNAs. At 48 h after transfection, cell lysates were analyzed using western blot with anti-FLAG antibody. The β-actin was used as a control. Lane headings showed the FLAG-tagged Ago1 isoforms and the isoform-specific siRNAs. The Ago1A/B-siRNA could specifically target both Ago1A and Ago1B. The antibodies used were indicated on the left.

### RNAi Assays

To silence the expression of shrimp Ago1 isoforms, sequence-specific siRNAs consisting of 21-nt double-stranded RNAs, the antisense strand of which contained a 19-nt target sequence and a two-uracil (U) overhang at the 3′-end, were used in this study. Based on the different sequences of Ago1 isoforms, the siRNAs targeting various isoforms (Table S1) were synthesized. The Ago1A/B-siRNA targeting both Ago1A and Ago1B (Table S1) was included in the siRNA synthesis. As a control, the scrambled sequence of Ago1A siRNA was used as the control siRNA (Table S1). The siRNAs were synthesized *in vitro* using the In vitro Transcription T7 Kit for siRNA Synthesis (Takara) according to the manufacturer’s protocol. The formation of synthetic siRNAs was monitored by 2% agarose gel electrophoresis to ensure that dsRNAs migrated as a single band. The synthesized siRNAs were dissolved in phosphate-buffered saline (PBS) solution (0.1 M, pH 7.4). The RNA concentration was determined using a NanoDrop ND-100 spectrophotometer (NanoDrop Technologies, Wilmington, DE, USA) at 260 nm.

**Figure 6:**
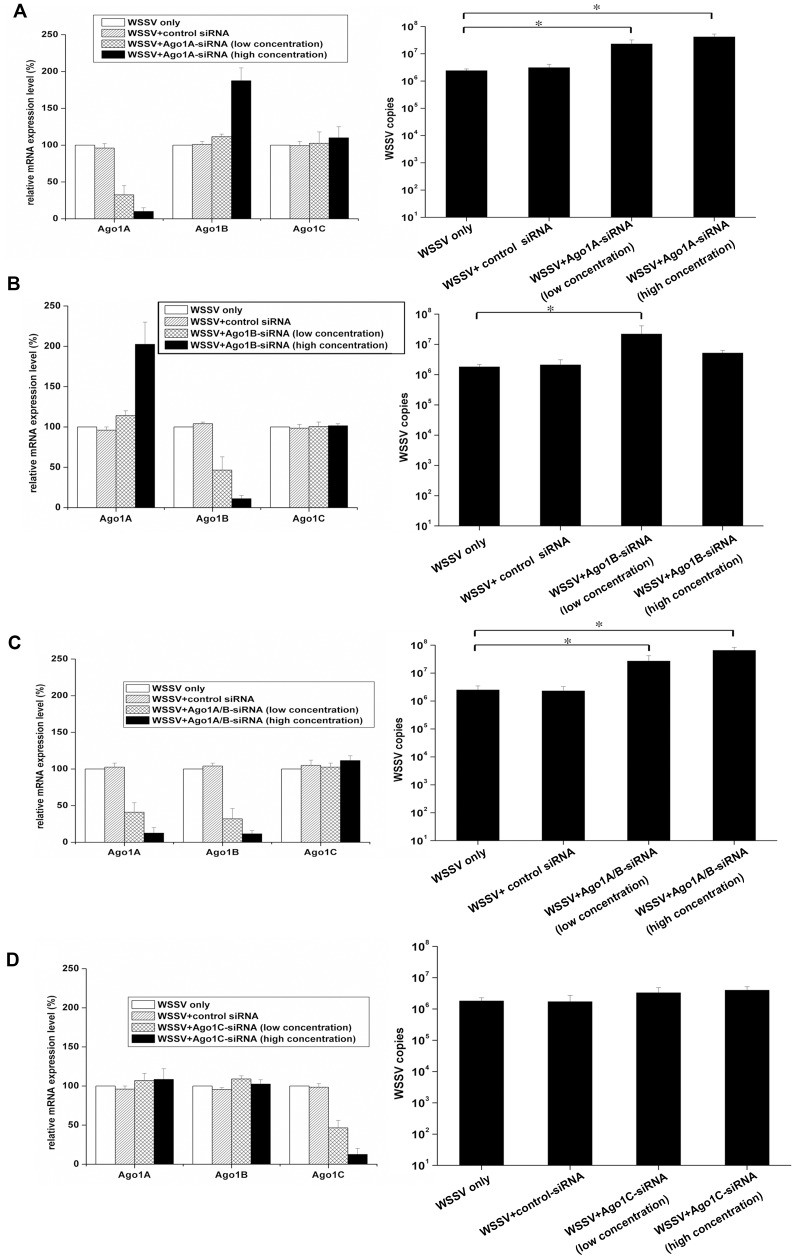
Roles of Ago1 isoforms in the shrimp immune response against WSSV infection. To characterize the roles of Ago1 isoforms in the antiviral immunity, shrimp were injected with WSSV and isoform-specific siRNAs. Shrimp were injected simultaneously with WSSV and the low or high concentration of Ago1A-siRNA (**A**), Ago1B-siRNA (**B**), Ago1A/B-siRNA (**C**) or Ago1C-siRNA (**D**), respectively. As control, WSSV+control siRNA and WSSV only were included in the injections. At 48 h post-inoculation, the shrimp from each treatment were subjected to quantitative real-time PCR to quantify the expressions of Ago1A, Ago1B, and Ago1C (left). The solutions used for injections were shown in the box. At the same time, the WSSV loads in shrimp were monitored by quantitative real-time PCR (right). The statistically significant differences between treatments were represented with asterisk (*P<0.05). Lane headings showed the solutions used for injections.

### Quantitative Real-time PCR (qRT-PCR)

The qRT-PCR assay was conducted using sequence-specific primers and TaqMan fluorogenic probes. The amplification of shrimp β-actin was used as a control. The primers and TaqMan probes used in the qRT-PCR were listed in Table S1. Reactions were prepared in a total volume of 25 µL containing 12.5 µL Premix Ex Taq (Takara), 1 µL cDNA template, 0.5 µL of 10 µM forward and reverse primers and 0.5 µL of 10 µM TaqMan fluorogenic probes to a final concentration of 0.2 µM. Amplification profiles consisted of 95°C for 1 min, and 40 cycles of 95°C for 15 s and 55°C for 45 s. Expression levels of Ago1 isoforms were normalized to those of shrimp β-actin.

To quantify WSSV in shrimp, qRT-PCR was conducted using WSSV-specific primers and a TaqMan fluorogenic probe (Table S1). The linearized plasmid containing a 1400-bp DNA fragment from the WSSV genome was used as an internal standard for qRT-PCR [19]. Virus genomic DNA was extracted from shrimp gills using SQ Tissue DNA Kit (Omega Bio-Tek, Norcross, GA, USA) according to the manufacturer’s instructions. The PCR mixture (25 µL) contained 12.5 µL Premix Ex Taq (Takara), 1 µL DNA template, 0.5 µL 10 µM forward and reverse primers and 0.5 µL 10 µM TaqMan fluorogenic probe at a final concentration of 0.2 µM. The reaction profile was 95°C for 1 min, followed by 45 cycles of 30 s at 95°C, 30 s at 52°C, and 30 s at 72°C.

### Southern Blot and Northern Blot Analysis

For Southern blot analyses, genomic DNA was prepared from shrimp gills using a SQ tissue DNA Kit (Omega Bio-Tek) according to the manufacturer’s protocols. The genomic DNA from shrimp lymphoid organs was digested with *Pvu*I or *EcoR*I. After separation by electrophoresis on a 1.0% agarose gel, the genomic DNA was detected using a digoxigenin (DIG)-labeled Ago1-probe that could detect three Ago1 isoforms or the Ago1-fragment 2-probe that was unique to Ago1A and Ago1B (Table S1).

For northern blot analyses, total RNA was extracted from the gills of shrimp and quantified using a spectrophotometer (NanoDrop Technologies). As a negative control, total RNA was treated with RNase A (Roche, Basel, Switzerland). Digested genomic DNA or total RNA (30 µg) was separated by electrophoresis on a 1.0% agarose gel in 1×TBE buffer (90 mM Tris-boric acid, 2 mM ethylenediaminetetraacetic acid; pH 8.0). The separated DNA or RNA was transferred to a Hybond-N+ nylon membrane (Amersham Biosciences, Buckinghamshire, UK). The membrane was pre-hybridized in DIG Easy Hyb buffer (Roche) for 0.5 h, followed by hybridization with a sequence-specific DIG-labeled probe at 55°C overnight. The Ago1-probe (Table S1) could detect three isoforms of Ago1. The Ago1-fragment 2-probe (Table S1) was used to detect Ago1A and Ago1B. Detection was performed using the DIG High Prime DNA Labeling and Detection Starter Kit II (Roche) following the manufacturer’s instructions.

### Cell Culture and Transfection

*Drosophila* Schneider 2 (S2) cells were propagated in *Drosophila* SDM (serum-free medium; Invitrogen, Grand Island, NY, USA) supplemented with 10% heat-inactivated fetal bovine serum (FBS) (PAA Laboratories, Linz, Austria). The PCR products of Ago1A, Ago1B or Ago1C tagged with the FLAG sequence were digested with *EcoR*I/*Xho*I and ligated to the pAc5.1/V5-His B (Invitrogen). Recombinant plasmids were confirmed by nucleotide sequencing. At approximately 70% confluence of S2 cells, 2 µg of Ago1A, Ago1B or Ago1C construct was co-transfected with 100 pmol of an isoform-specific siRNA or control siRNA (Table S1) using the Cellfectin II reagent (Invitrogen) according to the manufacturer’s instructions.

### Western Blot Assay

At 48 h after transfection, S2 cells were harvested and lysed in 0.4 mL of NP-40 lysis buffer (Sangon, Shanghai, China) containing protease inhibitors (Roche) on ice. After a 15 min centrifugation (14,000×*g*, 4°C), the aspirated supernatant was subjected to sodium dodecyl sulfate-polyacrylamide gel electrophoresis (SDS-PAGE) and transferred to a nitrocellulose membrane (Bio-Rad, Hercules, CA, USA). The membrane was immersed in blocking buffer [5% (w/v) skim milk and 0.1% (v/v) Tween-20 in PBS] at 4°C overnight, followed by incubation with anti-FLAG antibody (Invitrogen). Subsequently, the membrane was incubated in alkaline phosphatase (AP)-conjugated goat anti-mouse IgG (Sigma, St. Louis, MO, USA) for 1 h and detected with nitro-blue tetrazolium and 5-bromo-4-chloro-3′- indolyphosphate (NBT/BCIP) solutions (Sangon).

### *In vivo* Analysis

Shrimp were simultaneously injected with WSSV virions (10^4^ copies/shrimp) and either 20 µg (low concentration) or 30 µg (high concentration) of one of the siRNAs (Ago1A-siRNA, Ago1B-siRNA, Ago1C-siRNA and Ago1A/B-siRNA) (Table S1). Negative control shrimp were injected with WSSV virions (10^4^ copies/shrimp) and 30 µg of control siRNA. Positive control shrimp were injected with WSSV only (10^4^ copies/shrimp). For each treatment, a group of 10 individual shrimp were used and gill tissues were collected at 48 h post-inoculation. Three shrimp specimens were selected at random from each treatment and were subjected to qRT-PCR to quantify the mRNA levels of all three Ago1 isoforms and WSSV genome copies.

### Statistical Analysis

The numerical data from three independent experiments was analyzed by one-way analysis of variation (ANOVA) to calculate the mean and standard deviation. Statistical significance between treatments was carried out using the Student’s *t*-test.

## Results

### Identification of Ago1 Isoforms in Shrimp

Based on PCR amplification using degenerated primers and RACE analysis, full-length cDNA of the shrimp Ago1 gene was obtained. Sequence analysis revealed that the Ago1 gene generated three transcripts: Ago1A (GenBank accession no.: GU265732), Ago1B (GenBank accession no.: JX170715) and Ago1C (GenBank accession no.: JX170716). Sequence comparisons showed that the Ago1 isoforms were different from each other with a 9-nt inserted fragment (Ago1-fragment 1) at their 5′ termini and an additional 81-nt fragment of (Ago1-fragment 2) in the PIWI domain (Fig. 1). No amplification was observed when the extracted RNA was used in PCR analyses. These findings indicated that the Ago1 transcripts amplified here were not generated from genomic DNA (data not shown).

Multiple sequence alignments of shrimp Ago 1 isoforms (Ago1A, Ago1B, and Ago1C), Ago1, and Ago2 from other species revealed that the Ago1 sequences of *M. japonicus* shrimp were more closely related to Ago1 sequences of other animals, including humans, than to other animal Ago2 sequences (Fig. 2). The amino acid sequences of shrimp Ago1A, Ago1B, and Ago1C were almost identical, but differed at their N-terminal regions and PIWI domains (Fig. 1 & Fig. 2). An insertion of 27 amino acid residues was found in Ago1A and Ago1B isoforms, but not in the Ago1C isoform and Ago1 homologs of other species (Fig. 1 & Fig. 2), indicating that the 27-amino-acid domain might be unique in shrimp.

To exclude the possibility that Ago1 isoforms were generated from various copies of the Ago1 gene in the shrimp genome, Southern and northern blot analyses were conducted. Southern blots showed a single band (Fig. 3A), suggesting that there was one copy of Ago1 in the shrimp genome. Northern blot analysis revealed that two bands were hybridized with the Ago1 probe that could detect all the three isoforms of Ago1 (Fig. 3B). Meanwhile, only one band equivalent to the upper band was detected using the Ago1-fragment 2 probe that was unique to Ago1A and Ago1B isoforms, which presumably co-migrate because of similar molecular weights (Fig. 3B). These data indicated that the Ago1 isoforms were transcribed from the same Ago1 gene and might result from alternative splicing of the Ago1 precursor mRNA.

### Expression Patterns of Ago1 Isoforms in Shrimp

To characterize the expression patterns of Ago1 isoforms in different organs of shrimp, qRT-PCR was conducted using isoform-specific primers and probes. The isoform Ago1C was shown to be ubiquitously expressed in all organs or tissues examined (Fig. 4A). However, the mRNA levels of both Ago1A and Ago1B were significantly up-regulated in lymphoid organ (Fig. 4A), suggesting that the two isoforms were involved in shrimp immunity.

Considering that Ago proteins represent key components in antiviral RNAi immunity in many species [12], [13], [14], [15], the effects of WSSV infection on the expression of Ago1 isoforms was investigated. Because of the up-regulation of Ago1A and Ago1B isoforms in shrimp lymphoid organs, the lymphoid organ was selected to investigate the expression profiles of Ago1 isoforms in response to WSSV challenge. It was showed that the expression of Ago1A and Ago1B was significantly increased at 12 and 24 h post-inoculation (p<0.05) (Fig. 4B), whereas the expression of Ago1C remained unchanged at all time points compared to the control (0 h post-inoculation) (Fig. 4B). Taken together, these results indicated that Ago1A and Ago1B isoforms that contained the Ago1-fragment 2 played important roles in shrimp antiviral immunity.

### Effects of Ago1 Isoforms on Shrimp Antiviral Immunity

To investigate the roles of Ago1 isoforms in antiviral immunity, the expression of Ago1 isoforms were each silenced in shrimp using isoform-specific siRNAs, followed by WSSV challenge. First, to test the specificities of Ago1 isoform-specific siRNAs, FLAG-tagged Ago1 isoform constructs and isoform-specific siRNAs were transfected into S2 cells. Western blot analysis showed that the expression of Ago1A, Ago1B or Ago1C isoforms was inhibited by the corresponding sequence-specific Ago1A-siRNA, Ago1B-siRNA or Ago1C-siRNA, but not affected by control siRNAs and other isoform-specific siRNAs (Fig. 5). These data revealed that the Ago1A/B-siRNA targeting both Ago1A and Ago1B could silence the expression of both Ago1A and Ago1B, but not Ago1C (Fig. 5). Sequence analysis indicated three nucleotides were different between Ago1A and Ago1C at the 5′ termini (Fig. 1). Western blotting revealed that the Ago1A-siRNA could not knockdown the expression of Ago1B and Ago1C, and the Ago1B-siRNA could not silence the expression of Ago1A and Ago1C (Fig. 5). These data showed that the siRNAs used here were highly sequence- specific.

It was found that the expression of endogenous Ago1A was knocked down by approximately 55–80% by Ago1A-siRNA at the low concentration, resulting in an 11-fold increase of viral loads compared with the control (WSSV only) (P<0.05). However, the control siRNA at the high concentration had no effect on the Ago1A expression and virus replication (Fig. 6A). Interestingly, when Ago1A-siRNA was injected at high concentration, Ago1A mRNA was reduced by 85–95% and the Ago1B mRNA was significantly up-regulated at the same time (Fig. 6A).

Using these conditions, WSSV infection in shrimp was evaluated. Near-complete knockdown of Ago1A led to approximately 20-fold increase in viral load in the treatment (WSSV+ Ago1B-siRNA [high concentration]) compared with the control (WSSV only) (P<0.05) (Fig. 6A), indicating that Ago1A played an important role in WSSV infection. To inhibit the expression of Ago1B, Ago1B-siRNA was delivered at low or high concentration into shrimp, followed by the evaluation of WSSV infection in shrimp. It was demonstrated that Ago1B mRNA was reduced by 30–63% when shrimp were injected with Ago1B-siRNA at the low concentration, leading to a 12-fold increase in WSSV loads compared with the control (WSSV only) (P<0.05) (Fig. 6B). These data suggested that Ago1B was also involved in the host defense against virus infection. However, the near-complete inhibition of Ago1B expression by Ago1B-siRNA at high concentration also induced a significant up-regulation of the Ago1A mRNA, but no significant difference in viral loads was observed between treatment (WSSV+Ago1B-siRNA [high concentration]) and the control (WSSV only) (Fig. 6B). These data suggested that the up-regulation of Ago1A might compensate for the loss of Ago1B in the host defense against WSSV infection.

In contrast to the antiviral roles of the up-regulation of Ago1A, the up-regulation of Ago1B could not compensate for the loss of Ago1A for inhibiting viral replication (Fig. 6A & B). Thus, Ago1A and Ago1B might be involved in distinct pathways for defense against WSSV infection. To simultaneously silence the expressions of endogenous Ago1A and Ago1B isoforms, Ago1A/B-siRNA was injected into shrimp at low concentration that resulted in a significant increase (approximately 15-fold, P<0.05) in WSSV copies (Fig. 6C). In particular, the reduction of Ago1A and Ago1B mRNAs by Ago1A/B-siRNA at high concentration led to an approximate 26-fold increase of viral loads in WSSV-infected shrimp compared with the control (WSSV only) (P<0.05) (Fig. 6C). The simultaneous inhibition of Ago1A and Ago1B by Ago1A/B-siRNA resulted in a greater increase in viral loads than Ago1A or Ago1B alone. These results showed that Ago1A and Ago1B likely play important roles in the host defense against virus infection. As shown in Fig. 6D, the Ago1C isoform did not affect WSSV replication. Thus, overall, it could be concluded that Ago1A and Ago1B isoforms were involved in the host immune response against virus infection, suggesting a novel role of Ago isoforms in shrimp antiviral immunity.

## Discussion

Ago proteins, the effector molecules of siRNA and miRNA pathways, play crucial roles in RNAi and are involved in many physiological processes. In recent years, many Ago proteins and isoforms have been characterized. However, the roles of Ago isoforms are not clear. The present study showed that there were three isoforms of Ago1 (Ago1A, Ago1B and Ago1C) in shrimp. Sequence alignments indicated that Ago1 sequences of *M. japonicus* displayed higher sequence similarities to Ago1 proteins than Ago2 proteins of other species. Our study, together with a previous report of the identification of the *Litopenaeus vannamei* Ago1 and Ago2 [20], suggested that shrimp Ago1 protein likely played a role in miRNA-mediated gene silencing, while shrimp Ago 2 protein was potentially involved in siRNA-mediated antiviral defense. Our study showed that most sequences of the three isoforms were identical, but differed at their N-terminal region flanking the PAZ and PIWI domains. As reported, Ago proteins play important roles in host innate antiviral immunity [12], [13], [14], [15]. Therefore, the contributions of Ago1 isoforms to the antiviral immunity of shrimp were evaluated. The results indicated that Ago1A and Ago1B, which contained an additional 81-nt fragment (Ago1-fragment 2) in the PIWI domain, affected the shrimp immune response against WSSV infection. Given the key roles of Ago proteins in the host defense against viruses, it is proposed that the isoforms of Ago might be involved in the fine-tuning of host antiviral responses.

It is well known that suppressors of RNAi are widely expressed by viruses to counteract host RNAi immunity. Ago proteins, key components of antiviral RNAi pathways, are likely to represent hotspots of host-virus interactions. In this context, the sequence diversification of Ago1 proteins (Ago1 isoforms) might be a consequence of host adaptive evolution in response to viral threats, which was preserved in shrimp during long-term host-pathogen interactions. Similar to our findings, it was revealed that *A. gambiae* mosquitoes can employ alternative splicing of Down syndrome cell adhesion molecule (Dscam) immunoglobulin to generate an extremely diverse set of more than 31,000 potential alternative splice forms, which enables mosquito-specific recognition and defense against a broad spectrum of pathogens [21]. It was shown that different pathogen taxa induce pathogen challenge-specific splice form repertoires in the adult mosquito [21]. Recently, multiple Ago2 mRNA variants with two types of GRRs in the N-terminal portion were demonstrated in *Drosophila* [16], [17], where the GRRs of the long Ago2 isoform were shown to be essential for the normal function of the protein. An altered number of GRRs in long Ago2 isoform causes defects in RNAi and embryonic development that results in disruption of the midblastula transition from membrane growth to microtubule-based organelle transport [16]. However, the short Ago2 isoform with variant GRR copy numbers do not impair RISC assembly and support normal development [17]. The variation observed in N-terminal domain of short Ago2 isoform in *Drosophila* remains unclear [17]. In this context, the Ago isoforms revealed in this study represent an important strategy of host immune system to fight against virus invasion.

As demonstrated in the present study, the Ago1A and Ago1B isoforms containing Ago1 fragment 2 provide the molecular basis for the shrimp antiviral defense. To our knowledge, our study was the first report on the roles of Ago isoforms that might be generated by alternative splicing from a single gene in host immunity against virus infection in invertebrates. Invertebrates might have evolved alternative splicing strategies to generate functionally different isoforms to fine-tune the host antiviral responses.

In our study, Ago1A and Ago1B were shown to be involved in host immune responses against WSSV. It was revealed that the knockdown of Ago1B by a low concentration of siRNA-Ago1B significantly increased viral loads after virus challenge, suggesting that Ago1B was involved in the host defense against virus infection. However, the silencing of Ago1B by siRNA-Ago1B at the high concentration resulted in up-regulation of Ago1A and the simultaneous up-regulation of Ago1A could compensate for the loss of Ago1B in the shrimp defense against WSSV infection. Furthermore, knockdown of Ago1A by siRNA-Ago1A at the high concentration led to a significant increase in WSSV copies, although the Ago1B mRNA levels were also up-regulated, suggesting that the up-regulation of Ago1B could not compensate for the depletion of Ago1A in shrimp antiviral immunity. Therefore, it could be inferred that the Ago1 isoforms (Ago1A and Ago1B) might be involved in different pathways to control WSSV replication in shrimp. The mechanism for the compensatory regulation of different Ago isoforms in the host antiviral immunity warranted further investigation.

Overall, our study described the presence of three isoforms of the Ago1 protein in shrimp (*M. japonicus*) and investigated the roles of the different isoforms in antiviral shrimp response upon WSSV challenge. Silencing Ago 1A or Ago 1B significantly increased virus load compared to control shrimp (WSSV challenged only), indicating that Ago1A and Ago1B might play important roles in the host defense against virus infection. In contrast, silencing Ago 1C did not affect virus load, indicating that this isoform has no significant antiviral role. This study provided new insights into understanding the role of Ago 1 protein in antiviral response in invertebrates.

## Supporting Information

Table S1 Primers, probes and siRNAs used in this study.
(DOC)
