# Supplementary material for: Contribution of the Argonaute-1 Isoforms to Invertebrate Antiviral Defense
Source: PLoS One. 2012 Nov 29;7(11):e50581. doi: 10.1371/journal.pone.0050581 (PMC3510085; doi:10.1371/journal.pone.0050581)
Supplement: Table S1 — Primers, probes and siRNAs used in this study. (DOC) [file pone.0050581.s001.doc]

| Name | Sequence (5’-3’) |
| --- | --- |
| **primers** |  |
| WSSV-specific forward primer | TATTGTCTCTCCTGACGTAC |
| WSSV-specific reverse primer | CACATTCTTCACGAGTCTAC |
| Ago1 degenerate forward primer | GGDATGGCMACYCARTGTGT |
| Ago1 degenerate reverse primer | GTRCCCTGDATGCCCTGRTG |
| Ago1 5’RACE primer 1 | CCGCTGGTGGATGAGTTACA |
| Ago1 5’RACE primer 2 | CGCCTTTCGGACCAGATTGT |
| Ago1 5’RACE primer 3 | AATAGCCATCTGGAGCCGAG |
| Ago1 3’RACE primer 1 | TGTAACTCATCCACCAGCGG |
| Ago1 3’RACE primer 2 | TATCGGGATGGTGTGAGCGA |
| Ago1 full-length forward primer | AGGAGTAGAAATCAGTCTCGCA |
| Ago1 full-length forward primer | GAGGCAGACTAGGTAAGGAGAG |
| Ago1A forward primer | CCAGAACTACCTAACACA CCAG |
| Ago1A reverse primer | TGCCGTCCTCGCGAAAGTCAG |
| Ago1B forward primer | ACCGCCAG AACTACCTACT |
| Ago1B reverse primer | TGCCGTCCTCGCGAAAGTCAG |
| Ago1C forward primer | CC AGAACTACCTAACACACCTT |
| Ago1C reverse primer | CTGGATCACCTCCTGTCTGTGC |
| β-actin forward primer | CGAGCACGGCATCGTTACTA |
| β-actin reverse primer | TTGTAGAAAGTGTGATGCCAGATCT |
| WSSV forward primer | TTGGTTTCATGCCCGAGATT |
| WSSV reverse primer | CCTTGGTCAGCCCCTTGA |
| **Probes** |  |
| Ago1A TaqMan probe | FAM-CCGGGTCCAGCAGCACAGACAG-TAMRA |
| Ago1B TaqMan probe | FAM-CCAGTGACGGCTCGCGACCC-TAMRA |
| Ago1C TaqMan probe | FAM-AGGTGGAAGAGAAGTGTGGTTTGGC-TAMRA |
| β-actin TaqMan probe | FAM-CTGGGACGACATGGA-TAMRA |
| WSSV TaqMan probe | FAM-TGCTGCCGTCTCCAA-TAMRA |
| DIG-labeled Ago1 probe | GTATGGGTCATTGTTGAAGTCCGCCT |
| DIG-labeled Ago1-fragment2  probe | CACTGGCACTTTGGCCTTGTGTTGTTGATC |
| **siRNAs** |  |
| Ago1A-siRNA | ACTACCTAACACACCAGCT |
| Ago1B-siRNA | ACCGCCAGAACTACCTACT |
| Ago1C-siRNA | GACAGGAGGTGATCCAGGA |
| Ago1A/B-siRNA | TCAACAACACAAGGCCAAA |
| control siRNA | TAACCAAACCAGAGCACAA |

(R= A/G; M=A/C; Y=C/T; D=A/G/T)
